# Equity, Equal Shares or Equal Final Outcomes? Group Goal Guides Allocations of Public Goods

**DOI:** 10.3389/fpsyg.2017.00036

**Published:** 2017-01-25

**Authors:** Ali Kazemi, Daniel Eek, Tommy Gärling

**Affiliations:** ^1^School of Health and Education, University of SkövdeSkövde, Sweden; ^2^Department of Psychology, University of GothenburgGöteborg, Sweden

**Keywords:** group goal, public good dilemma, social dilemma, public good provision, allocation, equity, equal shares, equal final outcomes

## Abstract

In an experiment we investigate preferences for allocation of a public good among group members who contributed unequally in providing the public good. Inducing the group goal of productivity resulted in preferences for equitable allocations, whereas inducing the group goals of harmony and social concern resulted in preferences for equal final outcomes. The study makes a contribution by simultaneously treating provision and allocation of a public good, thus viewing these as related processes. Another contribution is that a new paradigm is introduced that bears closer resemblance to real life public good dilemmas than previous research paradigms do.

## Introduction

Individuals in groups often choose between acting selfishly or cooperatively. Social dilemma research investigates this tension between individual and collective rationality. Specifically, a social dilemma is a mixed-motive conflict in which members of a group decide whether to maximize the own interest or the interest of the group. The dilemma is that, no matter how other members of the group choose, it is more attractive for each individual to choose an alternative that increases his or her selfish interest (i.e., to defect) rather than the group’s interest (i.e., to cooperate). But if all members do this, all will receive a poorer outcome than if everyone chooses to cooperate (Dawes, 1980).

In this paper we focus on *public good dilemmas*, referring to that group members through individual contributions realize or maintain a common resource from which all can benefit. Game theory assumes that people are rational and selfish. Thus, in public good dilemmas, the rational choice is to *not* contribute to the public good because non-contributors (i.e., defectors) will also benefit from the good if provided. However, since the good can only be realized through sufficient contributions from members of the group, all will receive less if no one contributes than if all group members do. Consequently, considering the *group’s interest*, the rational choice is to contribute to the good’s provision.

Previous theory and research on public good dilemmas have predominantly focused on determinants of cooperation, that is, how much each group member contributes to the public good (e.g., Chen and Komorita, 1994). In a fruitful line of research, the importance of fairness for willingness to cooperate has been highlighted (e.g., Wilke, 1991; Kerr, 1995; Van Lange and Messick, 1996; Eek and Biel, 2003). In general, this research suggests that people contributes to public goods to the extent that they consider doing it as fair.

We argue that previous research has tended to neglect that public good dilemmas entail two processes: willingness to provide the public good and preferences for how the public good is allocated. Since these processes may not be independent of each other, it is important to simultaneously examine both. In the present study we do this by investigating what is a preferred allocation of a public good to group members who collectively have provided the public good, and how these allocation preferences vary with the goal the group has.

### Allocation Preferences

Theory and research has focused on several allocation principles (e.g., Kazemi and Törnblom, 2008; Törnblom and Kazemi, 2015, see also Törnblom and Kazemi, 2012a). According to *equity*, also referred to as the proportionality principle, the outcome for an individual should be proportional to his or her contribution. *Equal shares* refers to that individuals receive the same outcome regardless of possible differences in, for instance, the size of their contributions or personal need of the resource. According to *equal final outcomes*, all individuals should receive the same amount of the resource after it has been allocated. Thus, the allocation takes into account the state before the allocation, that is, each individual’s initial assets or needs (e.g., Van Dijk et al., 1999).

Deutsch (1975) argued that equity is associated with effectiveness and productivity. The argument is that, if an individual who has the capacity to use a limited resource in the most efficient way receives a greater share from the resource, it will lead to growth of the public good and, consequently, to the realization of the group goal. In contrast, whenever enjoyable social relations and harmony are dominant group goals, equality (equal shares) is likely to be applied because it enhances the egalitarian social standing among members of the group. Need-based allocations are endorsed in situations where the focus is individuals’ welfare, a sense of concern and responsibility for others. If initial differences in needs exist an allocation principle that achieves equal final outcomes would eliminate these differences.

We conjecture that group goals define desired future states. Attaining these states is why a collective resource is allocated on the basis of a given principle (Weber et al., 2004). Tenbrunsel and Messick (1999) argued that the impact of sanctions on cooperation is dependent on an activated frame (business vs. ethical). In a related vein, people tend to cooperate more in social dilemmas involving non-economic outcomes (i.e., contributing to a social event) than in social dilemmas involving economic outcomes (i.e., investing in a joint investment fund) (Pillutla and Chen, 1999). Although these studies are relevant in that they are concerned with the role of context and frames for decision making, they do not address the issue the present study raises of how allocation preferences vary with group goal.

### The Public Good Dilemma Paradigm

Two aspects of our public good paradigm deserve attention. First, in contrast to previous public good paradigms (e.g., Van Dijk and Wilke, 1995), we did not specify that the good would be divided equally among the group members. Thus, when deciding how much to contribute, participants were uncertain about how the public good following a successful provision of the public good would be divided among the members. In fact, in real-life public good dilemmas, people seldom know how public goods will be allocated. For instance, tax payers are uncertain about how the accumulated tax pool will be used. Thus, citizens do not know how much benefits they would receive. Second and related to the first aspect, the public good dilemma paradigm was extended to allow for other principles than equality to be endorsed in the allocation of public goods. In investigating the effects of group goal on public good allocations, participants were told that they in their positions as leaders would not receive anything from the public good. Consequently, contrary to how a public good is conventionally defined, non-excludability did not apply in the allocation of the public good. However, we argue that our public good simulation has a closer resemblance to real life. An example of a real-life public good dilemma is making contributions to charities (e.g., Cornes and Sandler, 1996). It is evident that non-excludability does not apply in this case, and that there usually is an individual or a group of individuals deciding how the charities are allocated to the needy.

Another aspect of the non-excludability issue in public good dilemmas has to do with whether real public goods are public in the sense that they are accessible to all people. Following Foddy (2005), restricting or excluding people’s access to scarce public goods is a common structural solution that governmental agencies employ. Health care, voting, immigration policy, and drivers’ licenses are all examples of this type of solution showing that public goods are provided only for some “publics” that in a way qualify for benefiting from the public good. Foddy concludes that the publics who contribute to the provision and the publics who benefit from its provision may therefore vary. Thus, given that real-life public goods are amenable to allocations based on other principles than equal share, such as equity and equal final outcomes, we believe that the present research addresses an important point that has been overlooked in previous research. Moreover, as Messick (1995) notes, although there is consensus about using equality in terms of the equal shares principle in the allocation of public goods, problems may still arise. Specifically, the type of resource to be allocated is important (Törnblom and Kazemi, 2012b). Messick discusses the problem of identifying ways to allocate an expensive oriental carpet between two persons who have equal claims to the carpet. It is obviously easier to divide equally a continuous resource such as money. Thus, real-life public goods cannot always be allocated equally because “public” does not mean “accessible to all people” (Foddy, 2005) and because public goods differ with regard to the nature of the resource that constitutes them (Messick, 1995). In conclusion, the present study contributes to experimental public good dilemma research as the newly introduced paradigm mimics real-life public goods in a way that has not been done in previous research.

### Aim and Hypotheses

The aim of this study is to examine the effects of group goal on preferred allocations of a public good. Inducement of the group goals of economic productivity, harmony, and social concern are hypothesized to affect the preferred allocation of the public good. Specifically, participants who are asked to encourage economic productivity are expected to prefer equity, participants who are asked to encourage harmony are expected to prefer equal shares, and participants who are asked to encourage social concern are expected to prefer equal final outcomes.

Major and Deaux (1982) distinguished between four types of allocation paradigms: allocations-to-others-only, allocations-to-self-only, individual allocations-to-self-and-others, and group allocations-to-self-and-others. In order to control for potential selfish influences on allocation preferences, we used the allocations-to-others-only paradigm according to which participants made allocations in the role of a randomly chosen group leader. Furthermore, we used a step-level symmetric public good paradigm. Symmetric indicates that all participants initially have an equal number of endowments to contribute, and the step-level feature denotes that the public good is provided only if a certain provision threshold is reached by the contributions, and that additional contributions do not increase the value of the public good.

## Methods

### Participants

Fifty three female undergraduates with a mean age of 23.8 years and seven male undergraduates with a mean age of 25.3 years were compensated with a movie pass for participating in the study. Their ages ranged from 19 to 45 years. An equal number of participants (*n* = 20) were randomly assigned to one of three group goal conditions representing economic productivity, social concern, or harmony. Men were represented in all conditions.

### Procedure and Materials

Participants who agreed to participate were upon arrival seated in separate cubicles and were given a questionnaire with written instructions. All participants were informed that they belonged to a group consisting of five members. This information was not possible to question since the participants could not see each other. In order to also create a sense of interdependence and group identification, the first page in the questionnaire informed the participants that they were going to participate on different occasions, and that this was the first of three occasions. It was explained that on this first occasion the aim was to study how people make decisions in groups without communicating with each other. All communication between group members was therefore to be made through the experimenter.

Thereafter, the public good dilemma was introduced. The instructions read (translated from the Swedish): “You are part of a five-person group. One person will be selected as the leader of the group. The group’s first task is to open a joint account and try to gain interest on your money. Each of you has SEK 60. Individually you must decide how much you want to contribute to the account. You may contribute as much as you like (SEK 0–60). If you jointly contribute at least SEK 120, the group receives an interest of SEK 120. The balance of the account will then equal SEK 240. The leader will divide this amount between the other four members. The balance can never exceed SEK 240. If you jointly contribute less than SEK 120, the group will not receive the interest and contributions will not be given back to the contributors. Write on the line below how much you want to contribute.”

At this stage the participants were informed that their compensation for participating was contingent on whether the group had received the interest and how it would be allocated among them. After the contributions had been made they were informed that a leader whose task was to make the allocation would be appointed through a random procedure. In fact, all participants were bogusly told that they had been chosen to be the leader. They were further informed that the threshold of SEK 120 had successfully been reached such that the interest (public good) had been provided. In order to allocate the public good between the other group members, the leader was informed about their contributions (this information was bogus and the same for all, see **Table 1**), that is, one group member contributed nothing, another SEK 20, a third SEK 40, and the fourth SEK 60.

**Table 1:** Information about the public good presented to participants.

	Group member				
	A	B	C	D	Sum
Initial endowments	60	60	60	60	240
Contribution	0	60	20	40	120
Post-contribution possession	60	0	40	20	120
Equity	0	120	40	80	240
Equal shares	60	60	60	60	240
Equal final outcomes	30	90	50	70	240

*The words equity, equal shares, and equal final outcomes were never mentioned to the participants. Instead they were referred to as Principles A, B, and C.*

The group goal was introduced subsequently. For the goal of *economic productivity* the instructions read (translated from the Swedish): “Your group has a long-term goal of economic productivity. Hence, economic profit is the primary driving force. The emphasis is on measuring achievements with precision.” For *harmony* the goal description read: “Your group has a long-term goal of harmony. Hence, maintenance of enjoyable relations is the primary driving force. The emphasis is on enhancing the group spirit and fellowship.” For *social concern* the goal description read: “Your group has a long-term goal of social concern. Hence, giving help and support to fellow group members is the primary driving force. The emphasis is on being considerate and taking responsibility for other members.”

This was followed by the leader’s task of allocating the public good (SEK 240) between the four group members guided by the three principles of equity, equal shares, or equal final outcomes. Participants’ allocations constituted the main dependent variable. As a guide for their allocation decisions, the participants were presented a table (similar to **Table 1**) showing the other group members’ initial endowments, their contributions, their post-contribution possessions, and how the distribution could be made based on the three principles. Participants were also informed that these principles merely served as examples and that they may distribute the public good in any way they preferred. On a line below the information provided, the participants wrote the amounts they allocated to each group member. The participants made only one allocation, but as in **Table 1** they were shown all three allocation principles.

We operationalized equity with reference to participants’ past performance (i.e., contributions). Equity thus rendered all group members an amount proportional to their contributions. Equality rendered all group members the same share of the public good. Equal final outcomes were operationalized as an allocation leaving all group members with the same end states. They thus all received an amount that was inversely related to the amount they had left after they had contributed.

A male experimenter monitored participants. After completing the questionnaire, which required approximately 25 min, the experimenter informed the participants that the study was over, that there would be no other occasions in the future, and that the compensation for participating was the same for all participants regardless of the decisions they made during the experiment. They were thanked and paid a movie pass (equivalent to approximately USD 11.00).

### Research Ethics

The experiment was carried out in accordance with the recommendations of the Swedish Research Council’s ethical principles for the humanities and social sciences. Specifically, research participants were informed about the purpose of this research and consent for participation was acquired from them. Moreover, it was made clear that participation was voluntary and the collected data would be used for research purpose only and that the data would be stored in such a way that prevents unauthorized persons from having access to them.

## Results

### Contribution Decisions

Participants’ contributions ranged from SEK 20 to SEK 60, with a mean of SEK 44.9 (*SD* = 14.1). Contributions did not differ between the experimental groups. Only one participant contributed an equal share (one-fifth) of the provision threshold. Twenty four participants chose to contribute all, 16 contributed half, and 10 participants contributed two-thirds of their endowments.

### Allocation Preferences

Participants’ mean preferred allocations to group members A–D in each group goal condition are given in **Table 2**. Allocation preferences were not significantly correlated with contributions (*r*_equity_ = -0.11; *r*_equal shares_ = 0.07; *r*_equal final outcomes_ = -0.05).

**Table 2:** Means and Standard deviations of allocations related to group goal.

		Group member			
		A	B	C	D
Economic productivity	*M*	18.3	103.5	46.4	71.9
	*SD*	22.2	22.1	8.3	9.9
Harmony	*M*	26.0	94.0	48.5	71.5
	*SD*	13.5	13.5	4.9	4.9
Social concern	*M*	40.5	79.5	52.5	67.5
	*SD*	22.4	22.4	6.4	6.4

In order to construct a measure of allocation preference for the three proposed principles of equity, equality, and equal final outcomes, three difference scores were computed as the sum of the absolute difference between participants’ allocations to each of the group members A–D and the corresponding allocations according to the principles. For instance, the sum difference score from the equity principle was obtained by subtracting participants’ allocations from 0 for A, from 120 for B, from 40 for C, and from 80 for D, and then summing the absolute differences. Thus, the smaller the sum difference scores for the principle, the closer the allocations are to this principle.

Means of the sum difference scores are displayed in **Table 3** as well as the frequencies of participants who exactly followed any of the allocation principles. As highlighted by the italicized values in the table, the allocations were closest to equity for economic productivity and closest to equal final outcomes for both harmony and social concern. It may also be noted that 52 participants (87%) made allocations exactly following one of the principles. A χ^2^-test showed that for those participants the association between goal condition and allocation principle was significant, χ^2^(4) = 18.63, *p* = 0.001.

**Table 3:** Means and standard deviations of sum difference scores and frequencies of preferred allocation principle related to group goal.

Group goal		Allocation principle		
		Equity	Equal shares	Equal final outcomes
Economic productivity	*M*	*49.5*	111.8	62.8
	*SD*	59.3	58.7	29.8
	*f*	*9*	3	3
Harmony	*M*	69.0	91.0	*17.0*
	*SD*	36.4	36.4	29.9
	*f*	3	1	*14*
Social concern	*M*	106.0	60.0	*42.0*
	*SD*	55.5	51.1	44.0
	*f*	2	7	*10*

*The lower the difference score, the smaller is the deviation from a specified allocation principle. *f* refers to the frequency of participants who exactly followed each allocation principle (i.e., no difference) in each group goal condition. Italicized values indicate the three lowest sum difference scores.*

Three separate one-way analyses of variance (ANOVAs)^1^ on the sum difference scores were conducted to test the effect of group goal on allocation preference according to the three principles. Group goal was significant in each ANOVA, *F*(2,57) = 6.24, *p* = 0.004, ω^2^ = 0.15 (equity), *F*(2,57) = 5.51, *p* = 0.006, est. ω^2^ = 0.13 (equal shares), and *F*(2,57) = 8.47, *p* < 0.001, ω^2^ = 0.27 (equal final outcomes). The difference from equity was significantly smaller in the economic productivity condition than the average difference from equity in the other two conditions, *t*(57) = -2.70, *p* = 0.009. The difference from equal shares was significantly smaller in the social concern condition than the average difference from equal shares in the other goal conditions, *t*(57) = -3.05, *p* = 0.004. Finally, the difference from equal final outcomes was significantly smaller in the harmony condition than the average difference from equal final outcomes in the other goal conditions, *t*(57) = -3.67, *p* < 0.001^2^.

## Discussion

The present study of preferences for allocation of public goods complements previous research investigating public good provision (e.g., Eek and Biel, 2003). Furthermore, the results provide compelling evidence for the impact of group goal on the preferred allocations. This is in support of the conjecture that group goals are viewed as decision frames such that they exert an influence on allocation preferences (cf. Pillutla and Chen, 1999; Weber et al., 2004).

The hypothesis that allocations according to equity are preferred when groups are motivated to realize economic productivity was supported. However, contrary to the hypothesis, inducing the group goal of harmony made participants allocate the public good according to equal final outcomes as they, consistent with the hypothesis, also did when the group goal was social concern. We believe that these findings partly contrary to our hypotheses may tentatively be reconciled with our initial line of reasoning. Specifically, since equal final outcomes equalize positions at the end, it is still an allocation that may increase harmony in the group at the same time as it may reflect social concern by taking into account initial contributions to the public good. Yet, this does not support the contention that if equal final outcomes imply that individual contributions are neglected, social cohesion and harmony in the group will be impaired (Deutsch, 2000). In the present study in which participants were similar to each other, this fact may have played a larger role than their individual contributions. Not taking into account that different participants did not contribute equally much may thus have been in the interest of maintaining harmony in the group. Perhaps in the role of leader the participants imagined some legitimate justification for the differences in contribution such as inequality in ability to contribute. Some participants may also when acting as leaders themselves have belonged to those participants that contributed less than the others.

In an experimental setting, Austin (1980) showed that while college roommates disregarded individual differences in performance and divided a collective resource equally, strangers took individual differences in performance into consideration and made equitable divisions. Similarly, Eek et al. (2001) reported that equity was perceived as fairer than equality in privately provided child care, whereas in child care provided by the municipality equality was perceived as fairer. As collectives such as roommates and municipalities presumably are more concerned with relational goals (i.e., harmony and social concern) than performance-related goals (i.e., economic productivity), previous findings are consistent with our results in showing that collective goals guide allocation decisions. An important implication is that implementation of a given allocation principle may be justified in one context but not in another (cf. Mannix et al., 1995).

The proposed relationships between group goal and allocation preference in this research bears close resemblance to Fiske’s (1992) work on relational models. Fiske (1992) distinguished between four basic types: communal sharing, authority ranking, equality matching, and market pricing. As each type has its own norms of conduct, these schemas guide social behavior. Thus, when a relationship is conceived in terms of communal sharing, what is exchanged is not determined on the basis of what each individual has contributed. Instead, the particular resource is shared by all its members according to their *need*. In communal sharing relationships the goal of *social concern* is salient. When a relationship is conceived in terms of authority ranking, individuals receive benefits based on their input in terms of status and rank. Thus, the higher your rank in that particular relationship, the more you receive from the collective resource. When relationships are primarily defined in terms of market pricing, exchanges are mainly based on what people are able to pay or the principle of proportionality (i.e., equity). The more you contribute, the more you are entitled to receive. In market pricing relationships, the goal of *(economic) productivity* is salient. Finally, in the equality matching mode reciprocity and equality are emphasized. In this mode people do not share a collective resource in accordance with their need or input or rank, they share the resource equally. In equality matching relationships, the goal of *harmony* is salient. In sum, Fiske’s (1992) work suggests that in different types of social relationships people seem to pursue different types of goals. Thus, it could be argued that the induced group goals in the present research may have activated participants’ view of the type of relationship their group belonged to and consequently affected their preferred allocations.

It should also be noted that the salience of a goal guiding actions may differ depending on a group’s development phase or the faced social situation. In support of this view, Goode (1978) maintained that a single justice principle does not prevail in social groups because they have a dominant goal orientation. For instance, sororities (or fraternities) as “solidarity-oriented groups” may not in all of their activities endorse equality or consider it as fair or appropriate. Instead, Goode claimed that different allocation norms apply to different phases or situations in a group’s life rather than being based on a single norm always being dominant in guiding the group’s actions. In real-life settings there usually is a mixture of different goals at some point in time which makes it difficult, if not impossible, to apply a certain principle as being conducive to realizing a particular goal. Often a combination of different allocation principles is needed to operate side by side. Deutsch (2000) maintained that all three goals are important in a social system and that the allocation principles can be implemented in a way that is either mutually supportive or mutually inconsistent. He concluded that “it is a delicate balance that often tilts too far in one direction or the other” (p. 43).

Choice of allocation principle did not have any impact on participants’ own outcomes. Thus, participants acted as non-recipient allocators. This could be regarded as a limitation. We maintain, however, that the major aim of the present research was to investigate the “pure” effects of group goal on preferred allocations. Toward this end, we deliberately removed the self-interest component from the allocation task. It may still be argued that having a leader making the allocation introduces a threat to the external validity of the results. Nevertheless, we do not believe that this aspect of the paradigm change our conclusions about the role of group goal for allocation preferences. In addition, we stress that our research participants, prior to being informed that they had been chosen as the leader of their group, were in a public good dilemma conflict, and that they were informed that the outcome was contingent upon their contribution decisions. Still, future research should more closely investigate the interplay between the three motives of self-interest, fairness and group goal. We also note that the unfortunate dominance of women in our sample is another threat to the external validity of the results. It is not implausible that women are more sensitive to group goals in their allocation preferences than men would be. This is however another issue to be addressed in future research.

Taken together, the present study makes three contributions to public good dilemma research. The first lies in the simultaneous treatment of public good provision and allocation, thus conceiving these as related processes. Second, a new paradigm resembling real life public good dilemmas was introduced. Third, as group goals and resource allocation are integral parts of all social groups, knowledge in advance of how to distribute collective resources in order to realize these goals are essential to group functioning and effectiveness. The present results corroborated our line of reasoning in showing that the preferred allocation of benefits depends on group goal. This is in line with prior research showing that decision frames have an impact on how people behave in social dilemmas (e.g., Pillutla and Chen, 1999; Tenbrunsel and Messick, 1999). An important implication is that preferred allocations of public goods may not primarily depend on what contributions initially have been made but on decision frames (in terms of future group goals once made salient), for example, harmony, leading to the endorsement of equal final outcomes. Hence, how benefits are allocated to group members is crucial for the degree to which one believes that different group goals are attained.

## Author Contributions

AK has made the most contributions to all stages of the present research. DE and TG have mainly each made substantial contributions to the experimental design and preparation of the manuscript.

## Conflict of Interest Statement

The authors declare that the research was conducted in the absence of any commercial or financial relationships that could be construed as a potential conflict of interest.

## Footnotes

1 Two-way factorial ANOVAs were also performed including as the additional factor whether participants contributed all their endowments (1) versus only part of it (-1). No significant effects at *p* < 0.05 of the contribution factor were observed.

2 Additional analyses revealed that the difference from equity was significantly smaller in the harmony condition than in the social concern condition, *t*(38) = 2.49, *p* = 0.017, and that the difference from equal final outcomes was marginally smaller in the social concern condition than in the economic productivity condition, *t*(38) = 1.75, *p* = 0.089.
